# Extensive resection improves overall and disease-specific survival in localized anorectal melanoma: A SEER-based study

**DOI:** 10.3389/fsurg.2022.997169

**Published:** 2022-08-30

**Authors:** Chang Liu, Cuiping Tang, Jianbo Zhang, Peng Zhu

**Affiliations:** ^1^ Department of Gastrointestinal Anorectal Surgery, The Second Affiliated Hospital of Chongqing Medical University, Chongqing, China; ^2^ Department of cancer center, The Second Affiliated Hospital of Chongqing Medical University, Chong, China

**Keywords:** anorectal melanoma, local resection, extensive resection, localized cases, survival

## Abstract

**Background:**

Anorectal melanoma is a rare tumor with a dismal prognosis. The only promising treatment for anorectal melanoma is surgery, either extensive resection (ER) or local excision (LE). However, the optimal extent of resection is still controversial. The purpose of this study was to investigate whether the survival outcomes of anorectal melanoma at different stages are influenced by the surgical approaches (LE or ER) using the National Institute of Health's Surveillance, Epidemiology, and End Results Program (SEER) database.

**Methods:**

The Surveillance, Epidemiology and End Results (SEER) database was queried to identify patients treated for anorectal melanoma (2000–2018). Overall survival (OS) and disease-specific survival (DSS) outcomes were compared for the two surgical approaches (ER or LE) stratified by stage (localized, regional and distant).

**Results:**

A total of 736 patients were included in the study. Details of previous surgical procedures were available for 548 of the study patients: 360 (65.7%) underwent LE, and 188 (34.3%) underwent ER. In localized cases, 199 underwent LE, and 48 underwent ER. The OS (median 45 vs. 29 months, 5-year rate 41.7% vs. 23.4%) and DSS (median 66 vs. 34 months, 5-year rate 51% vs. 30.7%) of patients undergoing ER were significantly better (*p* = 0.009 and 0.041, respectively) than those who received LE. Multivariate analysis showed that the type of surgery was an independent prognostic factor for both OS and DSS. Among the regional cases, 89 cases had LE, and 96 cases had ER. Patients with regional disease who underwent ER had no significant differences in OS (23 vs. 21 months; *p* = 0.866) or DSS (24 vs. 24 months; *p* = 0. 907) compared to patients who underwent LE. In distant cases, 72 cases had LE, and 44 cases had ER. Patients with metastatic disease who had ER also had similar OS (median 11 vs. 8 months; *p* = 0.36) and DSS (median 11 vs. 8 months; *p* = 0.593) to those who underwent LE.

**Conclusion:**

Extensive resection can improve the long-term prognosis of localized anorectal melanoma compared to local excision, but the prognosis of the two surgical techniques is comparable in both regional patients and distant patients.

## Introduction

Anorectal melanoma is a rare disease with poor prognosis due to early metastasis, and its incidence has been continuously increasing over time (1, 2). Anorectal melanoma accounts for 0.4% to 1.6% of all malignant melanomas, 23.8% of all mucosal melanomas, and 1% of all anorectal malignant tumors, making it the third most prevalent site for melanoma after the skin and eyes (3–5). The symptoms of anorectal melanoma, on the other hand, are ambiguous and can be found in a variety of anorectal illnesses, such as hemorrhoidal disease, polyps, and squamous cell carcinoma, thereby delaying its diagnosis and treatment (6–8). The overall survival of anorectal melanoma remains dismal, with a 5-year overall survival (OS) rate ranging from 10% to 20% (1, 9).

Because of its low incidence, experience in the surgical treatment of anorectal melanoma is still limited. Surgical resection, either extensive resection (ER) or local excision (LE), is the most promising treatment for anorectal melanoma, providing the opportunity for long-term survival (10–12). However, the optimal extent of resection is still controversial. Several recent studies reported comparable survival outcomes between ER and LE for the treatment of locoregional anorectal melanoma (13, 14). In contrast, some earlier studies found that abdominoperineal resection provided a significantly longer survival than LE (15, 16).

The abovementioned studies did not distinguish between localized and regional patients; therefore, the conclusions might not be suitable for all anorectal melanoma patients. Due to the low overall incidence and difficulty of early detection, localized cases of anorectal melanoma are generally rare. Currently, only a handful of studies with small sample sizes have been conducted to verify the prognostic impact of various surgical types on anorectal melanoma at various stages, and contradictions exist in these studies (16, 17). The current study aims to investigate whether the survival outcomes of anorectal melanoma at different stages are influenced by the surgical type (LE or ER) using the National Institute of Health's Surveillance, Epidemiology, and End Results Program (SEER) database.

## Materials and methods

### Data collection and processing

**Figure 1:**
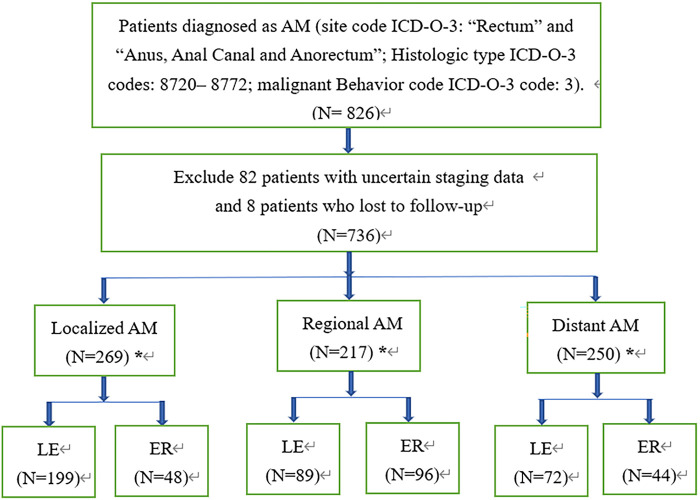
Flowchart for screening and statistical analysis of AM. *Stands for removing patients who have not undergone surgery, and the surgical method is unknown in the subsequent comparative analysis; AM, anorectal melanoma; LE, local excision; ER, extensive resection.

All patients diagnosed with melanomas of the anorectum were identified from 18 registries of the Surveillance, Epidemiology and End Results (SEER, http://seer.cancer.gov/) database between January 1, 2000, and December 31, 2018. The included patients satisfied the following criteria: anatomic sites of rectum or anus (site code ICD-O-3: “Rectum” and “Anus, Anal Canal and Anorectum”.); histologically diagnosed as malignant melanoma (Histologic type ICD-O-3 codes: 8720–8772, malignant Behavior code ICD-O-3 code: 3). The exclusion criteria were as follows: lost to follow-up, and unknown stage. Finally, a total of 736 patients with malignant melanoma were included in this study. The patients were divided into 3 groups by tumor stage according to the Clinical Combined Summary Stage developed for SEER: localized disease (stage I), regional disease (stage II, representing cases with regional lymph-nodal metastasis) and distant disease (stage III, referring to disease with distant metastasis). Each group was further divided into LE or ER subgroups according to the extent of surgery, and patients who did not undergo surgical resection or for whom the surgical method was unclear were excluded in this step (Figure 1).

In this study, LE includes “local tumor excision”, “polypectomy” and “excisional biopsy”. ER refers to the more extensive surgical approaches with pararectal lymph node removal, including “abdominal perineal resection”, “total proctectomy”, “total proctocolectomy” and “proctectomy”. Specifically, in rectal melanoma, cases with RX Summ Surg Prim Site (1998+) codes of 10–28 were identified as LE; in contrast, cases with Summ–Surg Prim Site (1998+) codes of 30–70 were categorized as ER. LE (Summ–Surg Prim Site codes: 10–27) and ER (Summ Surg Prim Site codes: 60–63) in anal melanoma were extracted similarly.

The survival data were calculated by SEER*Stat software using the survival session tool. In overall survival (OS), any death was considered an event, whereas in disease-specific survival (DSS), only death from the specific cancer was considered.

### Statistical analysis

SPSS version 25.0 (IBM Corp.) was used for statistical analysis and data management. Categorical variables are indicated as category names (number of cases, percentage), whereas continuous variables are indicated as the mean ± standard deviation or median. Chi-square analysis or Fisher's exact test was used to compare categorical data. Continuous variables were compared using the t test or Mann–Whitney test.

Survival curves were constructed using the Kaplan–Meier method, and the statistical significance of differences in survival was determined using the log-rank test. The Cox-hazard model was used for the univariate analysis and multivariate analysis. Variables with *p* < 0.1 in univariate analysis were included in multivariate analysis. The Wald backward method was used for multivariate analysis. A two-sided P value less than 0.05 indicated a significant difference. The SEER database is publicly available, and all patient information is deidentified; therefore, this study was granted an exemption from institutional review board approval.

## Results

### General features of patients

Among all 736 patients, 292 had rectal melanoma (39.7%), and 444 had anal melanoma (60.3%). The mean age was 67.9 ± 14.3 years, and the female-to-male ratio was 1.45:1. The number and proportion of cases with localized, regional and distant diseases were 269 (36.5%), 217 (29.5%) and 250 (34%), respectively. There were significant differences among the 3 groups in age, tumor location (rectum or anus), date of diagnosis, radiation, chemotherapy and surgery (Table 1).

**Table 1:** Characteristics of patients with different stages: localized, regional and distant.

	Localized	Regional	Distant	*p*
No. of patients	269	217	250	
Age(years)	69.5 ± 14.5	68.1 ± 13.3	65.9 ± 14.8	**0.016**
Sex				
Male	120 (44.6%)	84 (38.7%)	95 (38.0%)	0.245
Female	149 (55.4%)	133 (61.3%)	155 (62.0%)	
Location				
Rectum	105 (39.0%)	57 (26.3%)	130 (52.0%)	<**0.001**
Anus	164 (61.0%)	160 (73.7%)	120 (48.0%)	
Race				
White	228 (84.8%)	180 (82.9%)	213 (85.2%)	0.153
Black	16 (5.9%)	8 (3.7%)	18 (7.2%)	
Others	25 (9.3%)	29 (13.4%)	19 (7.6%)	
Date of diagnosis				
2000–2009	135 (50.2%)	105 (48.4%)	98 (39.2%)	**0.029**
2010–2018	134 (49.8%)	112 (51.6%)	152 (60.8%)	
Surgery				
Yes	248 (92.2%)	190 (87.6%)	124 (49.6%)	<**0.001**
No/unkonwn	21 (7.8%)	27 (12.4%)	126 (50, 4%)	
Radiation				
Yes	49 (18.2%)	33 (15.2%)	82 (32.8%)	<**0.001**
No/unkonwn	220 (81.8%)	184 (84.8%)	168 (67.2%)	
Chemotherapy				
Yes	24 (8.9%)	37 (17.1%)	81 (32.4%)	<**0.001**
No/unkonwn	246 (91.1%)	180 (82.9%)	169 (67.6%)	

### Survival analyses

Overall, patients with anorectal melanoma had a median OS of 17 months and a 5-year survival rate of 17%. Patients with localized disease had a median OS of 29 months and a 5-year survival rate of 25.6%, which were significantly superior to those of the regional (median OS 21 months, 5-year rate 16.1%; *p* = 0.013) and distant groups (median OS 7 months, 5-year rate 8.9%; *p* < 0.001).

Not surprisingly, patients who underwent surgical resection showed significantly longer OS (median 21 vs. 7 months; *p* < 0.001) and DSS (median 24 vs. 8 months; *p* < 0.001) than those who failed to undergo resection, irrespective of the stage classification. Furthermore, the stratified analyses based on tumor staging demonstrated that patients with both localized disease (median OS 31 vs. 12 months, *p* < 0.001; DSS 38 vs. 12 months, *p* < 0.001) and regional disease (median OS 22 vs. 12 months; *p* = 0.02; DSS 24 vs. 13 months; *p* = 0.024) could benefit from surgical treatment. However, for patients with distant metastases, surgical resection failed to show any statistically significant survival advantages (median OS 8 vs. 6 months, *p* = 0.051; DSS 8 vs. 6 months, *p* = 0.086), as shown in Table 2.

**Table 2:** Surgical Treatment and Survival by Stratified Analyses.

	NO	Surgery			No surgery			*p*
		NO	Median OS (95% CI)	Median DSS (95% CI)	NO	Median OS (95% CI)	Median DSS (95% CI)	
All	736	562	21.0 (18.1–23.9)		174	7.0 (5.2–8.8)		<0.001
				24.0 (20.3–27.7)			8.0 (6.0–10.0)	<0.001
Localized	269	248	31.0 (27.2–34.8)		21	12.0 (4.7–19.4)		<0.001
				38.0 (30.3–45.7)			12.0 (4.7–19.4)	<0.001
Regional	217	190	22.0 (18.8–25.2)		27	12.0 (75.2–16.8)		0.02
				24.0 (18.5–29.5)			13.0 (5.5–20.4)	0.024
Distant	250	126	8.0 (5.2–10.8)		124	6.0 (4.5–7.5)		0.051
				8.0 (5.2–10.8)			6.0 (4.0–8.0)	0.086

NO, Number of patients; OS, overall survival; DSS, disease-specific survival; 95% CI, 95% confidence interval.

### Survival benefit of different surgical extents for anorectal melanoma patients with various tumor stages

#### Localized disease group (stage I)

**Figure 2:**
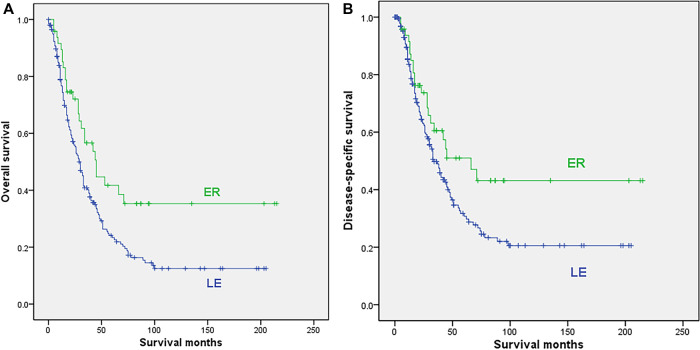
Survival curves for patients with localized disease who underwent local excision (LE) or extensive resection (ER); (**A**) overall survival; (**B**) disease-specific survival.

Among the 269 localized cases, 22 cases were excluded due to no surgery or unclear surgical method, and finally, 199 localized cases with LE and 48 cases undergoing ER were included in the analysis. Patients undergoing ER were younger (median 62.6 vs. 70.3 years, *p* = 0.001), while more patients in the LE subgroup received radiotherapy (21.6% vs. 6.3%, *p* = 0.014). The sex, tumor location, race, date of diagnosis and chemotherapy were comparable between the 2 subgroups, as shown in Supplementary Table S1. The survival analysis showed that ER was associated with significantly better OS (median 45 vs. 29 months, 5-year rate 41.7% vs. 23.4%; *p* = 0.009) and DSS (median 66 vs. 34 months, 5-year rate 51% vs. 30.7%; *p* = 0.041) than LE (Figure 2).

Only 137 of 199 cases in the LE subgroup and 35 of 48 patients in the ER subgroup had tumor size information, and the mean sizes were 31.3 mm and 31.7 mm, respectively, with no significant difference (*p* = 0.959). Seventeen patients undergoing LE and 32 patients in the ER subgroup had postoperative lymph node biopsy data, with average numbers of lymph node biopsies of 4.2 and 11.8, respectively. All lymph node biopsies were negative.

Univariable Cox regression analysis was conducted to determine the survival significance of these variables (sex, age, race, date of diagnosis, location, type of surgery, radiation, and chemotherapy), and the results indicated that RE was associated with better OS (HR = 0.579, *p* = 0.01) and DSS (HR = 0.626, *p* = 0.043) than LE. Multivariate analysis showed that the type of surgery, age and race were independent prognostic factors for OS, and the type of surgery and race were independent prognostic factors for DSS of localized anorectal melanoma patients (Table 3, Supplementary Table S4).

**Table 3:** Cox regression analysis of prognostic factors influencing OS for patients with localized disease.

	Univariable analysis			Multivariable analysis		
	HR	95% CI	*p*	HR	95% CI	*p*
Age(years)			< **0.001**	** **		**0**.**002**
<60	1			1		
60–74	1.511	(0.978–2.336)	0.063	1.375	(0.890–2.126)	0.151
≥75	2.256	(1.514–3.362)	<0.001	1.974	(1.323–2.947)	0.001
Sex			0.347			
Male	1					
Female	1.156	(0.853–1.567)				
Date of diagnosis						
Continuous	0.973	(0.944–1.003)	0.076			
2000–2009	1					
2010–2018	0.669	(0.485–0.922)				
Location			0.203			
Rectum	1					
Anus	0.818	(0.6–1.115)				
Race			**0**.**048**	** **		0.098
White	1			1		
Black	0.706	(0.346–1.440)	0.338	0.745	(0.365–1.522)	0.419
Others	0.515	(0.292–0.910)	0.022	0.511	(0.311–0.975)	**0**.**041**
Surgery			**0**.**01**	** **		**0**.**03**
LE	1			1		
ER	0.579	(0.381–0.880)		0.627	(0.411–0.957)	
Radiation			0.154			
No/unkonwn	1					
Yes	0.742	(0.492–1.120)				
Chemotherapy			0.804			
No/unkonwn	1					
Yes	1.067	(0.637–1.788)				

HR, hazard ratio; 95% CI, 95% confidence interval; LE, local excision; ER, extensive resection.

#### Regional disease group (stage II)

**Figure 3:**
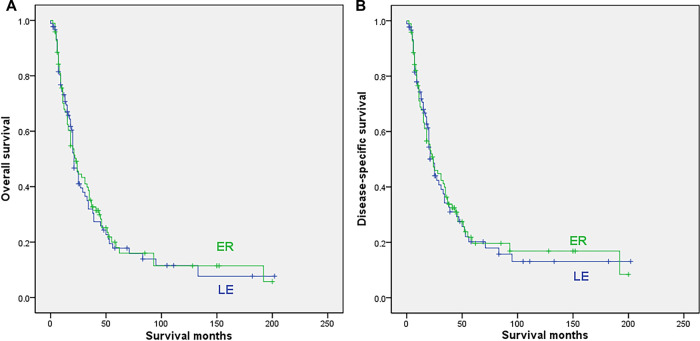
Survival curves for patients with regional disease who underwent local excision (LE) or extensive resection (ER); (**A**) overall survival; (**B**) disease-specific survival.

After excluding cases with no surgery or unclear surgical methods, 89 cases with LE and 96 cases undergoing ER were finally included in the analysis. Patients undergoing ER were also younger (median 65 vs. 69.6 years, *p* = 0.02). The sex, race, date of diagnosis, radiation and chemotherapy were all comparable between the 2 subgroups (Supplementary Table S2). The survival analysis indicated that the type of surgery showed no significant influence on either OS (median 23 months for ER vs. 21 months for LE, 5-year rate of 18% for ER vs. 17.8% for LE; *p* = 0.866) or DSS (median 24 months for both ER and LE, 5-year rate of 19.6% for ER vs. 20.2% for LE; *p* = 0. 907), as shown in Figure 3.

The univariable Cox regression analysis showed that patients receiving chemotherapy had a worse prognosis for OS (*p* = 0.02) and DSS (*p* = 0.014). Multivariate analysis showed that age and chemotherapy were independent prognostic factors for both the OS and DSS of regional anorectal melanoma patients (Table 4, Supplementary Table S5).

**Table 4:** Cox regression analysis of prognostic factors influencing OS for patients with regional disease.

	Univariable analysis			Multivariable analysis		
	HR	95% CI	*p*	HR	95% CI	*p*
Age(years)			**0** **.** **043**	** **		**0**.**017**
<60	1			1		
60–74	0.9	(0.591–1.370)	0.623	1.003	(0.652–1.542)	0.989
≥75	1.455	(0.956–2.216)	0.08	1.683	(1.084–2.612)	0.02
Sex			0.136			
Male	1					
Female	0.769	(0.543–1.088)				
Date of diagnosis						
Continous	0.98	(0.949–1.012)	0.216			
2000–2009	1		0.14			
2010–2018	0.77	(0.544–1.091)				
Location			0.411			
Rectum	1					
Anus	0.848	(0.572–1.256)				
Race			0.916			
White	1					
Black	1.189	(0.522–2.709)	0.68			
Others	0.992	(0.622–1.582)	0.975			
Surgery			0.868			
LE	1					
ER	0.972	(0.697–1.356)				
Radiation			0.23			
No/unkonwn	1					
Yes	0.739	(0.450–1.214)				
Chemotherapy		** **	**0.02**		** **	**0.007**
No/unkonwn	1			1		
Yes	1.636	(1.075–2.489)		1.831	(1.180–2.843)	

HR, hazard ratio; 95% CI, 95% confidence interval; LE, local excision; ER, extensive resection.

#### Distant disease group (stage III)

**Figure 4:**
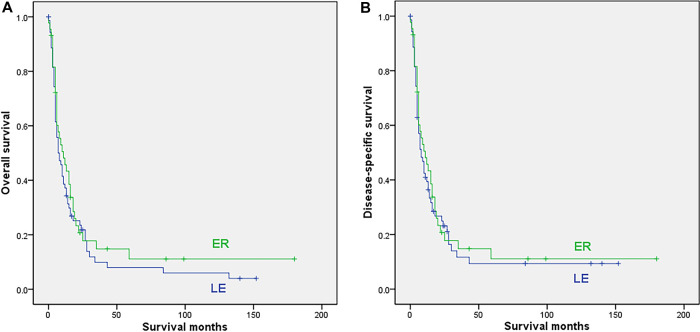
Survival curves for patients with distant disease who underwent local excision (LE) or extensive resection (ER); (**A**) overall survival; (**B**) disease-specific survival.

In this group, 72 cases with LE and 44 cases undergoing ER were finally included in the analysis. The mean age, location, race, date of diagnosis, radiation and chemotherapy were comparable between the 2 subgroups, while the proportion of females was significantly higher (*p* = 0.017) in the ER subgroup, as shown in Supplementary Table S3. The survival analysis showed that ER was not associated with either OS (median 11 vs. 8 months; 5-year rate not reached for ER vs. 7.9% for LE; *p* = 0.36) or DSS (median 11 vs. 8 months, 5-year rate not reached for ER vs. 9.4% for LE; *p* = 0.593) for patients with distant disease (Figure 4).

The univariable Cox regression analysis showed that older age and race, except white and black, were related to both worse OS (*p* = 0.002; *p* = 0.05) and DSS (*p* = 0.007; *p* = 0.04). Multivariate analysis showed that age was an independent prognostic factors for OS, and age, tumor location were independent prognostic factors for DSS in distant anorectal melanoma patients (Table 5; Supplementary Table S6).

**Table 5:** Cox regression analysis of prognostic factors influencing OS for patients with distant disease.

	Univariable analysis			Multivariable analysis		
	HR	95% CI	*p*	HR	95% CI	*p*
Age (years)		** **	**0.002**		** **	**0.003**
<60	1			1		
60–74	1.356	(0.839–2.194)	0.214	1.375	(0.850–2.225)	0.194
≥75	2.365	(1.444–3.874)	0.001	2.343	(1.431–3.838)	0.001
Sex			0.927			
Male	1					
Female	0.981	(0.652–1.477)				
Date of diagnosis						
Continous	0.971	(0.934–1.009)	0.126			
2000–2009	1		0.246			
2010–2018	0.79	(0.529–1.178)				
Location			0.072			0.085
Rectum	1			1		
Anus	1.461	(0.964–2.213)		1.442	(0.951–2.185)	
Race			0.13			
White	1					
Black	1.231	(0.566–2.678)	0.604			
Others	1.844	(1.001–3.395)	**0**.**05**	** **		
Surgery			0.376			
LE	1					
ER	0.831	(0.551–1.253)				
Radiation			0.173			
No/unknown	1					
Yes	1.367	(0.870–2.146)				
Chemotherapy			0.966			
No/unkonwn	1					
Yes	1.009	(0.671–1.516)				

HR, hazard ratio; 95% CI, 95% confidence interval; LE, local excision; ER, extensive resection.

## Discussion

The data of this SEER-based study indicated the dismal prognosis of anorectal melanoma, with a 5-year OS rate of only 17%. However, the 5-year survival rate was 25.6% in patients with localized disease, which was significantly better than those with regional or distant disease. Surgical resection was associated with a significant survival advantage for anorectal melanoma patients with either localized or regional disease, while this benefit was not seen for patients with distant metastases. Regarding different surgical types (LE or ER), no significant prognostic difference was found between the ER and LE subgroups in patients with either regional or distant disease. However, our results demonstrated that ER could significantly increase both OS and DSS in patients with localized anorectal melanoma when compared to LE.

In 1995, a retrospective study of 74 cases from a single institution found that abdominoperineal resection was associated with a higher chance of long-term survival for patients with localized anorectal melanoma, so the authors concluded that abdominoperineal resection should be considered in localized anorectal melanoma patients, especially those with smaller tumors and no evidence of nodal metastases (16). Some recent studies have drawn different conclusions. In 2021, Jutten et al. (17) reported their results of 71 localized anorectal melanoma patients and found no significant differences in survival between LE and ER procedures (25 vs. 21 months, *p* = 0.228). In the study, 10 of 48 patients (21%) who received LE originally finally underwent ER after a median of 4 months. In a recent meta-analysis, a total of 278 localized cases from 8 studies were analyzed, and the results showed no significant difference (OR = 1.30, 95% CI. 0.62 to 2.72, *p* = 0.49) in OS between the LE and ER groups (18). However, this study employed the odds ratio as a summary statistic for time-to-event outcomes, which has been questioned because not all patients had events, and the follow-up durations and individual patients in those studies were nonhomogeneous (19). Our study is by far the largest stratified study, and our results from 247 localized anorectal melanoma cases demonstrated that ER could significantly increase both OS and DSS (*p* = 0.009 and *p* = 0.041, respectively) compared to LE. Multivariate analysis showed that ER was an independent prognostic factor for OS (HR = 0.627; *p* = 0.03) and DSS (HR = 0.624; *p* = 0.044).

For patients with regional disease, although no survival difference was shown between the two surgery approaches, surgical treatment significantly improved both OS and DSS when compared to nonsurgical patients. A previous study found that metastasis to locoregional lymph nodes was a major prognostic factor (11). Theoretically, lymphadenectomy with extensive excision (ER) can help improve local control. However, consistent with all currently available studies, our results indicated that ER could not bring survival benefit for patients with regional anorectal melanoma (20–25). We believe this is totally due to the lack of effective systemic therapies for this disease. In our study, radiotherapy and chemotherapy both failed to improve the prognosis of patients. In recent years, the prognosis of many solid tumors has been significantly improved, which is mainly attributed to the progress of systemic therapy, including chemotherapy, targeted therapy and immunotherapy. With the help of effective systemic therapy, the role of surgery is to improve local cancer control. For anorectal melanoma, without the help of effective systemic therapy to ensure the overall prognosis, ER and lymphadenectomy have a very limited impact on survival, leading to a poor prognosis similar to that of patients undergoing LE.

For the same reason, patients with localized anorectal melanoma might benefit from ER. Currently, extensive surgery may be the only effective treatment that helps control occult regional metastasis, which could possibly have occurred beyond imaging and histological detection. Moreover, for patients with advanced (metastatic) disease, any extent of surgery could not improve the prognosis. Hopefully, in the near future, effective systematic treatments can be developed, so patients could be treated with a smaller extent of surgery to achieve a better prognosis. However, at present, it is still essential to individualize the surgical strategy for anorectal melanoma patients according to accurate preoperative staging.

Our study has several limitations. First, the SEER database lacks information on the resection margin status, recurrence, and postoperative complications, which have been reported to be linked to prognosis in previous studies (26–28). Second, this is a retrospective analysis based on the SEER database; hence, there will be some selection bias. In the localized and regional groups, for example, we discovered that patients who choose ER were younger; despite using multifactor analysis to compensate for the influence of age, this bias still persisted. Third, the results of this study lack external data validation. Therefore, a randomized controlled clinical study based on accurate preoperative staging is mandatory in the future.

In conclusion, anorectal melanoma is a rare tumor with a dismal prognosis. Currently, ER can benefit the prognosis of patients with localized disease but not for patients with regional disease. If effective systemic therapies could be developed in the future, the prognostic value of ER for patients with localized and regional metastases should be reassessed.

## Data Availability

The data analyzed in this study was obtained from the National Institutes of Health (NIH) National Cancer institute (NCI) Surveillance, Epidemiology, and End Results (SEER) Program, the following licenses/restrictions apply: To request access to the SEER Incidence Databases, users must register through either the Institutional or Non-Institutional Account option. Requests to access these datasets should be directed to SEER, https://seerdataaccess.cancer.gov/seer-data-access.
