# Supplementary material for: Extensive resection improves overall and disease-specific survival in localized anorectal melanoma: A SEER-based study
Source: Front Surg. 2022 Aug 30;9:997169. doi: 10.3389/fsurg.2022.997169 (PMC9468230; doi:10.3389/fsurg.2022.997169)
Supplement: Supplementary file 1 [file Table_1_v1.docx]

|  |  | Local excision | extensive resection | P |
| --- | --- | --- | --- | --- |
| No. of patients | | 199 | 48 |  |
| age(years) |  | 70.3±13.8 | 62.6±14.6 | **0.001** |
| sex |  |  |  |  |
|  | male | 92(46.2%) | 18(37.5%) | 0.275 |
|  | female | 107(53.8%) | 30(62.5%) |  |
| location |  |  |  |  |
|  | rectum | 71(35.7%) | 23(47.9%) | 0.117 |
|  | anus | 128(64.3%) | 25(52.1%) |  |
| race |  |  |  |  |
|  | white | 170(85.4%) | 39(81.3%) | 0.652 |
|  | black | 10(5.0%) | 4(8.3%) |  |
|  | others | 19(9.5%) | 5(10.4%) |  |
| date of diagnosis | |  |  |  |
|  | 2000-2009 | 104(52.3%) | 23(47.9%) | 0.589 |
|  | 2010-2018 | 95(47.7%) | 25(52.1%) |  |
| radiation |  |  |  |  |
|  | yes | 43(21.6%) | 3(6.3%) | **0.014** |
|  | no/unkonwn | 156(78.4%) | 45(93.8%) |  |
| chemotherapy |  |  |  |  |
|  | yes | 16(8.0%) | 4(8.3%) | 0.947 |
|  | no/unkonwn | 18392.0%) | 44(91.7%) |  |

table s1 . Characteristics of patients with localized disease undergoing local excision and radical resection
