# Supplementary material for: Extensive resection improves overall and disease-specific survival in localized anorectal melanoma: A SEER-based study
Source: Front Surg. 2022 Aug 30;9:997169. doi: 10.3389/fsurg.2022.997169 (PMC9468230; doi:10.3389/fsurg.2022.997169)
Supplement: Supplementary file 2 [file Table_2_v1.docx]

|  |  | Local excision | extensive resection | P |
| --- | --- | --- | --- | --- |
| No. of patients | | 89 | 96 |  |
| age(years) |  | 69.6±13.6 | 65±12.9 | **0.02** |
| sex |  |  |  |  |
|  | male | 35(39.3%) | 35(36.5%) | 0.688 |
|  | female | 54(60.7%) | 61(63.5%) |  |
| location |  |  |  |  |
|  | rectum | 12(13.5%) | 30(31.9%) | **0.004** |
|  | anus | 77(86.5%) | 66(68.8%) |  |
| race |  |  |  |  |
|  | white | 73(82.0%) | 77(80.2%) | 0.169 |
|  | black | 1(1.1%) | 6(6.3%) |  |
|  | others | 15(16.9%) | 13(13.5%) |  |
| date of diagnosis | |  |  |  |
|  | 2000-2009 | 44(49.4%) | 52(54.2%) | 0.52 |
|  | 2010-2018 | 45(50.6%) | 44(45.8%) |  |
| radiation |  |  |  | 0.087 |
|  | no/unkonwn | 74(82.0%) | 87(90.6%) |  |
|  | yes | 16(18.0%) | 9(9.4%) |  |
| chemotherapy |  |  |  | 0.973 |
|  | no/unkonwn | 74(83.1%) | 80(83.3%) |  |
|  | yes | 15(16.9%) | 16(16.7%) |  |

Table S2. Characteristics of patients with regional disease undergoing local excision and radical resection
