# Supplementary material for: Extensive resection improves overall and disease-specific survival in localized anorectal melanoma: A SEER-based study
Source: Front Surg. 2022 Aug 30;9:997169. doi: 10.3389/fsurg.2022.997169 (PMC9468230; doi:10.3389/fsurg.2022.997169)
Supplement: Supplementary file 3 [file Table_3_v1.docx]

Table S3. Characteristics of patients with distant disease undergoing local excision and radical resection

|  |  | Local  excision | extensive resection | P |
| --- | --- | --- | --- | --- |
| No. of patients | | 72 | 44 |  |
| age(years) |  | 65.9±14.5 | 63.2±12.9 | 0.339 |
| sex |  |  |  |  |
|  | male | 31(43.1%) | 9(20.5%) | **0.013** |
|  | female | 41(56.9%) | 35(79.5%) |  |
| location |  |  |  |  |
|  | rectum | 28(38.9%) | 15(34.1%) | 0.604 |
|  | anus | 44(61.1%) | 29(65.9%) |  |
| race |  |  |  |  |
|  | white | 60(83.3%) | 37(84.1%) | 0.913 |
|  | black | 4(5.6%) | 3(6.8%) |  |
|  | others | 8(11.1%) | 4(9.1%) |  |
| date of diagnosis | |  |  |  |
|  | 2000-2009 | 36(50%) | 23(52.3%) | 0.812 |
|  | 2010-2018 | 36(50%) | 21(47.4%) |  |
| radiation |  |  |  | 0.95 |
|  | no/unkonwn | 56(77.8%) | 34(77.3%) |  |
|  | yes | 16(22.2%) | 10(22.7%) |  |
| chemotherapy |  |  |  | 0.945 |
|  | no/unkonwn | 47(65.3%) | 29(65.9%) |  |
|  | yes | 25(34.7%) | 15(34.1%) |  |
