# Supplementary material for: Extensive resection improves overall and disease-specific survival in localized anorectal melanoma: A SEER-based study
Source: Front Surg. 2022 Aug 30;9:997169. doi: 10.3389/fsurg.2022.997169 (PMC9468230; doi:10.3389/fsurg.2022.997169)
Supplement: Supplementary file 4 [file Table_4_v1.docx]

Table S4. Cox regression analysis of prognostic factors influencing DSS for patients with localized disease

|  |  | Univariable analysis | | | Multivariable analysis | | |
| --- | --- | --- | --- | --- | --- | --- | --- |
|  |  | HR | 95% CI | P | HR | 95% CI | P |
| age(years) | |  |  | 0.097 |  |  |  |
|  | ＜60 | 1 |  |  |  |  |  |
|  | 60-74 | 1.458 | (0.927-2.294) | 0.103 |  |  |  |
|  | ≥75 | 1.596 | (1.031-2.471) | **0.036** |  |  |  |
| sex |  |  |  | 0.621 |  |  |  |
|  | male | 1 |  |  |  |  |  |
|  | female | 1.091 | (0.773-1.538) |  |  |  |  |
| date of diagnosis | |  |  |  |  |  |  |
|  | continuous | 0.962 | (0.929-0.996) | **0.028** | 0.967 | (0.933-1.001) | 0.058 |
|  | 2000-2009 | 1 |  |  |  |  |  |
|  | 2010-2018 | 0.605 | （0.419-0.874） |  |  |  |  |
| location |  |  |  | 0.078 |  |  | 0.091 |
|  | rectum | 1 |  |  | 1 |  |  |
|  | anus | 0.733 | (0.518-1.037) |  | 0.738 | (0.518-1.050) |  |
| race |  |  |  | **0.047** |  |  | **0.035** |
|  | white | 1 |  |  | 1 |  |  |
|  | black | 0.554 | (0.226-1.358) | 0.197 | 0.516 | (0.210-1.266) | 0.149 |
|  | others | 0.495 | (0.259-0.946) | **0.033** | 0.511 | (0.267-0.977) | **0.025** |
| surgery |  |  |  | **0.043** |  |  | **0.044** |
|  | LE | 1 |  |  | 1 |  |  |
|  | ER | 0.626 | (0.396-0.990) |  | 0.624 | (0.394-0.987) |  |
| radiation |  |  |  | 0.437 |  |  |  |
|  | no/unkonwn | 1 |  |  |  |  |  |
|  | yes | 0.863 | (0.555-1.344） |  |  |  |  |
| chemotherapy | |  |  | 0.353 |  |  |  |
|  | no/unkonwn | 1 |  |  |  |  |  |
|  | yes | 1.29 | （0.753-2.210） |  |  |  |  |

HR, hazard ratio; 95% CI, 95% confidence interval; LE, local excision; ER, extensive resection.
