# Supplementary material for: Extensive resection improves overall and disease-specific survival in localized anorectal melanoma: A SEER-based study
Source: Front Surg. 2022 Aug 30;9:997169. doi: 10.3389/fsurg.2022.997169 (PMC9468230; doi:10.3389/fsurg.2022.997169)
Supplement: Supplementary file 5 [file Table_5_v1.docx]

Table S5. Cox regression analysis of prognostic factors influencing DSS for patients with regional disease

|  |  | Univariable analysis | | | Multivariable analysis | | |
| --- | --- | --- | --- | --- | --- | --- | --- |
|  |  | HR | 95% CI | P | HR | 95% CI | P |
| age(years) | |  |  | 0.074 |  |  | **0.032** |
|  | ＜60 | 1 |  |  | 1 |  |  |
|  | 60-74 | 0.875 | (0.567-1.351) | 0.547 | 0.978 | (0.627-1.542) | 0.921 |
|  | ≥75 | 1.392 | (0.901-2.148) | 0.136 | 1.618 | (1.028-2.546) | 0.038 |
| sex |  |  |  | 0.248 |  |  |  |
|  | male | 1 |  |  |  |  |  |
|  | female | 0.808 | (0.562-1.160) |  |  |  |  |
| date of diagnosis | |  |  |  |  |  |  |
|  | continous | 0.978 | (0.946-1.011) | 0.197 |  |  |  |
|  | 2000-2009 | 1 |  | 0.104 |  |  |  |
|  | 2010-2018 | 0.743 | (0.519-1.065) |  |  |  |  |
| location | |  |  | 0.438 |  |  |  |
|  | rectum | 1 |  |  |  |  |  |
|  | anus | 0.856 | (0.578-1.268) |  |  |  |  |
| race |  |  |  | 0.853 |  |  |  |
|  | white | 1 |  |  |  |  |  |
|  | black | 1.266 | (0.555-2.888) | 0.576 |  |  |  |
|  | others | 1.026 | (0.635-1.656) | 0.918 |  |  |  |
| surgery | |  |  | 0.908 |  |  |  |
|  | LE | 1 |  |  |  |  |  |
|  | ER | 0.98 | (0.694-1.384) |  |  |  |  |
| radiation | |  |  | 0.252 |  |  |  |
|  | no/unkonwn | 1 |  |  |  |  |  |
|  | yes | 0.749 | (0.449-1.249） |  |  |  |  |
| chemotherapy | |  |  | **0.014** |  |  | **0.006** |
|  | no/unkonwn | 1 |  |  | 1 |  |  |
|  | yes | 1.699 | （1.106-2.612） | | 1.88 | （1.200-2.948） |  |

HR, hazard ratio; 95% CI, 95% confidence interval; LE, local excision; ER, extensive resection.
