# Supplementary material for: Extensive resection improves overall and disease-specific survival in localized anorectal melanoma: A SEER-based study
Source: Front Surg. 2022 Aug 30;9:997169. doi: 10.3389/fsurg.2022.997169 (PMC9468230; doi:10.3389/fsurg.2022.997169)
Supplement: Supplementary file 6 [file Table_6_v1.docx]

Table S6. Cox regression analysis of prognostic factors influencing DSS for patients with distant disease

|  |  | Univariable analysis | | | Multivariable analysis | | |
| --- | --- | --- | --- | --- | --- | --- | --- |
|  |  | HR | 95% CI | P | HR | 95% CI | P |
| age(years) | |  |  | **0.007** |  |  | **0.009** |
|  | ＜60 | 1 |  |  | 1 |  |  |
|  | 60-74 | 1.313 | (0.803-2.147) | 0.278 | 1.336 | (0.817-2.186) | 0.249 |
|  | ≥75 | 2.201 | (1.330-3.642) | 0.002 | 2.177 | (1.316-3.602) | 0.002 |
| sex |  |  |  | 0.968 |  |  |  |
|  | male | 1 |  |  |  |  |  |
|  | female | 1.009 | (0.662-1.537) |  |  |  |  |
| date of diagnosis | |  |  |  |  |  |  |
|  | continous | 0.969 | (0.932-1.008) | 0.119 |  |  |  |
|  | 2000-2009 | 1 |  | 0.16 |  |  |  |
|  | 2010-2018 | 0.746 | (0.459-1.124) |  |  |  |  |
| location |  |  |  | 0.033 |  |  | **0.038** |
|  | rectum | 1 |  |  | 1 |  |  |
|  | anus | 1.6 | (1.038-2.466) |  | 1.581 | (1.025-2.439) |  |
| race |  |  |  | 0.112 |  |  |  |
|  | white | 1 |  |  |  |  |  |
|  | black | 1.162 | (0.505-2.674) | 0.723 |  |  |  |
|  | others | 1.899 | (1.029-3.502) | **0.04** |  |  |  |
| surgery |  |  |  | 0.604 |  |  |  |
|  | LE | 1 |  |  |  |  |  |
|  | ER | 0.896 | (0.590-1.359) |  |  |  |  |
| radiation | |  |  | 0.186 |  |  |  |
|  | no/unkonwn | 1 |  |  |  |  |  |
|  | yes | 1.363 | (0.860-2.160） |  |  |  |  |
| chemotherapy | |  |  | 0.925 |  |  |  |
|  | no/unkonwn | 1 |  |  |  |  |  |
|  | yes | 0.98 | （0.644-1.490） | |  |  |  |

HR, hazard ratio; 95% CI, 95% confidence interval; LE, local excision; ER, extensive resection.
